# Reliability of the parameters of the power-duration relationship using maximal effort time-trials under laboratory conditions

**DOI:** 10.1371/journal.pone.0189776

**Published:** 2017-12-15

**Authors:** Christoph Triska, Bettina Karsten, Bernd Heidegger, Bernhard Koller-Zeisler, Bernhard Prinz, Alfred Nimmerichter, Harald Tschan

**Affiliations:** 1 Centre for Sport Science and University Sports, University of Vienna, Vienna, Austria; 2 Department of Life and Sport Science, University of Greenwich, Kent, United Kingdom; 3 Department of Exercise and Sport Science, LUNEX International University of Health, Exercise and Sports, Differdingen, Luxembourg; 4 Austrian Institute of Sports Medicine, Vienna, Austria; 5 Training and Sports Sciences, University of Applied Sciences, Wr. Neustadt, Austria; Sao Paulo State University, BRAZIL

## Abstract

The purpose of this study was to assess the reliability of critical power (CP) and the total amount of work accomplished above CP (*W´*) across repeated tests using ecologically valid maximal effort time-trials (TT) under laboratory conditions. After an initial incremental exercise test, ten well-trained male triathletes (age: 28.5 ± 4.7 years; body mass: 73.3 ± 7.9 kg; height: 1.80 ± 0.07 m; maximal aerobic power [MAP]: 329 ± 41 W) performed three testing sessions (*Familiarization*, *Test I* and *Test II*) each comprising three TT (12, 7, and 3 min with a passive recovery of 60 min between trials). CP and *W´* were determined using a linear regression of power vs. the inverse of time (1/t) (P = *W´* ∙ 1/t + CP). A repeated-measures ANOVA was used to detect differences in CP and *W´* and reliability was assessed using the intra-class correlation coefficient (ICC) and the coefficient of variation (CoV). CP and *W´* values were not significantly different between repeated tests (*P* = 0.171 and *P* = 0.078 for CP and *W´*, respectively). The ICC between *Familiarization* and *Test I* was *r* = 0.86 (CP) and *r* = 0.58 (*W´*) and between *Tests I* and *II* it was *r* = 0.94 (CP) and *r* = 0.95 (*W´*). The CoV notably decreased from 4.1% to 2.6% and from 25.3% to 8.2% for CP and *W´*, respectively. Despite the non-significant differences for both parameter estimates between *Familiarization*, *Test I*, and *Test II*, ICC and CoV values improved notably after the familiarization trial. Our novel findings indicate that for both, CP and *W´* a familiarization trial increased reliability. It is therefore advisable to familiarize well-trained athletes when determining the power-duration relationship using TT under laboratory conditions.

## Introduction

A reliable determination of critical power (CP) and the total amount of work accomplished above CP until task failure (*W´*) has long been a question of interest. Whilst CP represents a work rate that can be sustained for a long time without a continuous loss of metabolic (e.g. pH, phosphocreatine) and systemic (blood lactate concentration, ${\dot{\text{V}}\text{O}}_{2}$) homeostasis [1], *W´* is an equivalent for a finite amount of work that can be accomplished above CP [2, 3]. Originally, the determination of CP and *W´* requires 3 to 5 constant-power time-to-exhaustion trials (TTE) on a cycle ergometer, leading to exhaustion within 2–15 min [e.g. 4, 5–7]. However, TTE have no predefined endpoints and therefore are not comparable to the tasks athletes are confronted with during competition.

Although TTE provide reliable results for CP (*r* = 0.90–0.96) [8–10], *W´* has consistently shown to be less reliable across repeated tests (*r* = 0.64–0.84) [8–10]. It should be noted that small differences in time-to-exhaustion between repeated trials might alter the parameter estimates (in particular *W´*) [11, 12]. Therefore, TTE efforts should be used with caution when trying to detect small training induced changes in an athlete’s performance [13].

Fixed duration time-trials (TT) with a known endpoint are typically used when CP and *W´* are determined under field conditions [4, 6, 7, 12, 14]. TT are often described as an optimal approximation of real-world conditions and therefore, have a higher ecological validity compared to TTE [4–7, 14, 15]. In addition, TT were found to have a high test-retest reliability [16, 17] also when compared to TTE efforts [4, 18]. From a practical point, trained athletes are commonly accustomed to TT type efforts as this is the typical exercise modality in competitions. It is therefore recommended, to prefer TT over TTE when constructing the power-duration relationship [6, 15].

Hampson et al. [19] argued that during TT efforts, athletes are able to change the intensity according to perception of fatigue and motivation. Whilst intensity fluctuations add some variability to the measurement [13], Jeukendrup and Currell [20] debated that pacing is an inherent strategic component of real-world performance and therefore, is an integral part of performance tests. The only recent work suggesting an improved performance using TTE was performed just recently by Coakley and Passfield [21]. Comparing time-matched TTE with TT, a higher average power output (PO) for the 80% TTE resulted in significantly higher values for CP and significantly lower *W´* values compared to those derived from the TT. Despite this finding, it is currently unclear, if CP derived from TTE represent a sustainable intensity. As a result of the constant power profile during TTE, as opposed to power fluctuations during TT, pain, discomfort and peripheral fatigue might be delayed [22, 23], and therefore could increase mean PO.

When using TT for the determination of CP and *W´*, Galbraith et al. [15] and Karsten et al. [7] demonstrated a high reliability for critical speed (the mode equivalent of CP in running) and CP respectively using ecologically valid TT efforts in the field (coefficient of variation [CoV] = 1.3–2.0% [15]; CoV = 2.2–2.5% [7]). However, similar to TTE efforts both studies demonstrated poor reliability for TT determined values of *W´* [7, 15] (CoV = 9.8–18.4% [15]; CoV = 46.0–46.7% [7]). Karsten et al. [7] speculated that differences in environmental conditions (e.g. terrain, cadence) or in the seating position might have affected reliability of *W´*, whilst Galbraith et al. [15] found an increased reliability after a familiarization session.

In contrast, Triska et al. [12] and Black et al. [24] found non-significant differences and a significant correlation in *W´* between TTE and TT running and cycling using time/work-matching TTE and TT efforts. However, a high intra-individual variation did not allow the interchangeable use of *W´* [12].

When testing for CP and *W´*, even well-trained cyclists appear to require two familiarization sessions when using fixed-duration TT in the laboratory. This was demonstrated by Parker Simpson and Kordi [25] who found significantly lower CP values during testing sessions 1 and 2 compared to subsequent sessions. Interestingly, no differences were found for *W´* across all trials. The importance of familiarization trials is further corroborated by other studies, showing a smaller CoV after familiarization [14, 15]. Galbraith et al. [15] argued that altered pacing strategies can result in smaller CoV values post familiarization. The same authors demonstrated a poor reliability of *W´* (ICC *r* = 0.75 and CoV = 32.7%) even though participants were familiarized [14]. However, the duration of the respective predictive runs were not matched in the latter study, what has been shown to affect the parameter estimates [12]. The reason for the high day-to-day variation of *W´* is still unclear and questions on whether *W´* can be accurately determined using the power-duration relationship, and if the estimated *W´* equals ‘physiological’ *W´*, remains to be elucidated [12, 26, 27].

To date the reliability of TT determined CP and *W´* values has not been demonstrated in the laboratory. Given present findings for *W´* [7, 12, 14], familiarization, controlled conditions, and matched durations of respective trials might provide some further insight into this apparent conundrum of a low reproducibility of *W´*. Therefore, the aim of this study was to assess the reliability and potential learning effects when using TT efforts to determine CP and *W´* under controlled conditions. We hypothesized non-significant differences for CP and *W´*, a smaller CoV, and higher ICC after familiarization.

## Material and methods

### Participants

Ten well-trained male triathletes (age: 28.5 ± 4.7 years; body mass: 73.3 ± 7.9 kg; height: 1.80 ± 0.07 m; maximal aerobic power [MAP]: 329 ± 41 W) volunteered to participate in this study. All participants were involved in regular training and competition for at least three years on a national competition level and were experienced in performing TT. Before entering the study, participants completed a health questionnaire and provided written informed consent after the nature and risks of the study had been explained. The ethics committee of the University of Vienna (#00216) approved all experimental procedures and the study was conducted in accordance with the *Declaration of Helsinki*.

### Study design

The study followed a repeated laboratory test design where participants reported to the laboratory on four occasions separated by at least 72 h. A preliminary graded exercise test (GXT) was followed by three visits consisting of three TT each. These TT were between 3 and 12 min in duration and interspersed by 60 min passive rest to allow blood lactate [La] to return to baseline values in order to minimize any effect of prior exercise on ${\dot{\text{V}}\text{O}}_{2}$ uptake kinetics on the subsequent trial [5, 27]. Tests were performed at the same time of the day (± 2 h) in an air-condition controlled laboratory. Temperature and relative humidity were between 22–23°C and 45–55%, respectively. Participants were instructed to arrive at the laboratory in a fully hydrated state and to avoid strenuous exercise and alcohol intake in the 24 h prior to testing. Participants were also required to refrain from food and caffeine 3 h prior to testing. For all tests, a Cyclus2 ergometer (RBM Elektronics, Leipzig, Germany) was used where participants used their own racing or TT bike, which was mounted to the ergometer. During all tests, participants were strongly verbally encouraged. Testing was completed within 3 weeks to avoid effects of training and detraining. All tests were performed outside of the competitive season (i.e. during the participants’ off-season) during which each participant trained between 3 to 5 h per week. The majority of the participants completed the tests within 12–13 days, with the exception of a single participant who completed the tests within 16 days. However, in this single participant the GXT and the familiarization session were separated by 7 days and the two CP-tests were separated by 72 h.

### Graded exercise test

A GXT was performed to determine MAP. After an unloaded cycling phase for 3 min, resistance was set to 100 W and was increased by 20 W every 3 min until volitional exhaustion. If the last work stage could not be fully completed, MAP was calculated using the following equation of Kuipers et al. [28]:

$$\text{MAP}={\text{P}}_{\text{last}}+\left(\frac{\text{t}}{180}\cdot 20\right)$$ (1)

where MAP is the maximum aerobic power (W), P_last_ is the last fully completed work stage (W) and t is the duration of the incomplete work stage (s).

### TT to determine the power-duration relationship

Participants performed three identical tests to determine the power-duration relationship. The first test was used as a familiarization session and it was included in the analysis. The first test is consequently termed *Familiarization*, and the second and third test *Test I* and *Test II*, respectively. During the TT participants were advised to produce the highest mean power output for 12, 7 and 3 min in that order [29] and were instructed to complete each trial maximally (‘maximal TT effort’) [5]. Participants were able to manipulate their cadence and gear throughout the trials by using the virtual gear changer mounted to the handlebar thus simulating field-based TT. Moreover, participants used a self-selected pacing strategy. Transitions from rest to work were with an increase of pedal cadence to the participants’ own preferred value after a 3-min unloaded cycling phase. During the TT, PO increased as a function of cadence and pedal force.

### Estimation of CP and *W´*

Mean PO for each TT was plotted against the inverse-of-time using a linear regression where PO is the mean power output (W), *W´* is the total amount of work accomplished above CP until task failure (J) and CP is the critical power (W):

$$\text{PO}=W^{\prime }\cdot \frac{1}{\text{t}}+\text{CP}$$ (2)

Least square modelling procedures were used to fit the parameter estimates. The y-intercept represents CP and the slope represents *W´*. The individual SEE was calculated for each participant and each parameter estimate in absolute and relative values. Nimmerichter et al. [30] demonstrated that the model power vs. the inverse of time provides notably lower SEE compared to other two parameter models [30]. Analysing the parameter estimates of the three most commonly used models to estimate CP and *W´* (i.e. hyperbolic model of power vs. time, linear model of work vs. time, and linear model of power vs. inverse of time) revealed non-significant differences between the models, neither for CP (*P* = 0.353, *P* = 0.887, and *P* = 0.909 for *Familiarization*, *Test I* and *Test II*, respectively) nor for *W´* (*P* = 0.180, *P* = 0.867, and *P* = 0.812 for *Familiarization*, *Test I* and *Test II*, respectively). Consequently, we decided to use the model that provides the smallest error of the estimates (SEE) and thus results in most accurate estimates of CP and *W´* [30].

### Statistical analyses

After testing for normality using Shapiro-Wilk procedures, a repeated-measures analysis of variance (ANOVA) was conducted to assess differences between the tests. If the assumption of sphericity had been violated (*P* < 0.001) the Greenhouse-Geisser correction has been used [31]. Significant main effects were followed-up by Bonferroni post-hoc procedures. Partial eta-squared $\left(\eta_{p}^{2}\right)$ was used to provide an estimate of effect size of the ANOVA (small $\eta_{p}^{2}=0.01$; moderate $\eta_{p}^{2}=0.10$; large $\eta_{p}^{2}=0.25$). Effect size for the post-hoc tests was calculated using Cohen’s *d* (small *d* = 0.2; moderate *d* = 0.5; large *d* = 0.8) [32]. The intra-class correlation coefficient (ICC) and the coefficient of variation (CoV) were calculated using a spreadsheet [33]. An ICC >0.9 indicates *high* reliability, values >0.8 indicate *moderate* reliability, values >0.6 indicate *questionable* reliability, and values <0.6 indicate *poor* reliability of repeated tests. The coefficient of variation (CoV) was used to rate intra-individual variation. An upper limit of 5% [33] or 10% [34] is proposed to provide reliable results when repeating two tests. The Bland-Altman’s method of 95% limits of agreement (LoA) assessed the agreement between repeated tests for CP and *W´* [35]. Pearson product moment correlation assessed the strength of an association between repeated tests. Statistical significance was accepted at *P* < 0.05. Before the beginning of the study an *a priori* power-analysis was conducted and revealed that 10 participants were required to detect a significant difference of 15 W and 3 kJ for CP and *W´*, respectively with a statistical power of >80% [36]. A difference of 15 W in CP and 3 kJ in *W´* would result in a calculated TT_20min_ time difference of <5%. That is within the typical day-to-day variation of TT performance [12].

## Results

**Fig 1:**
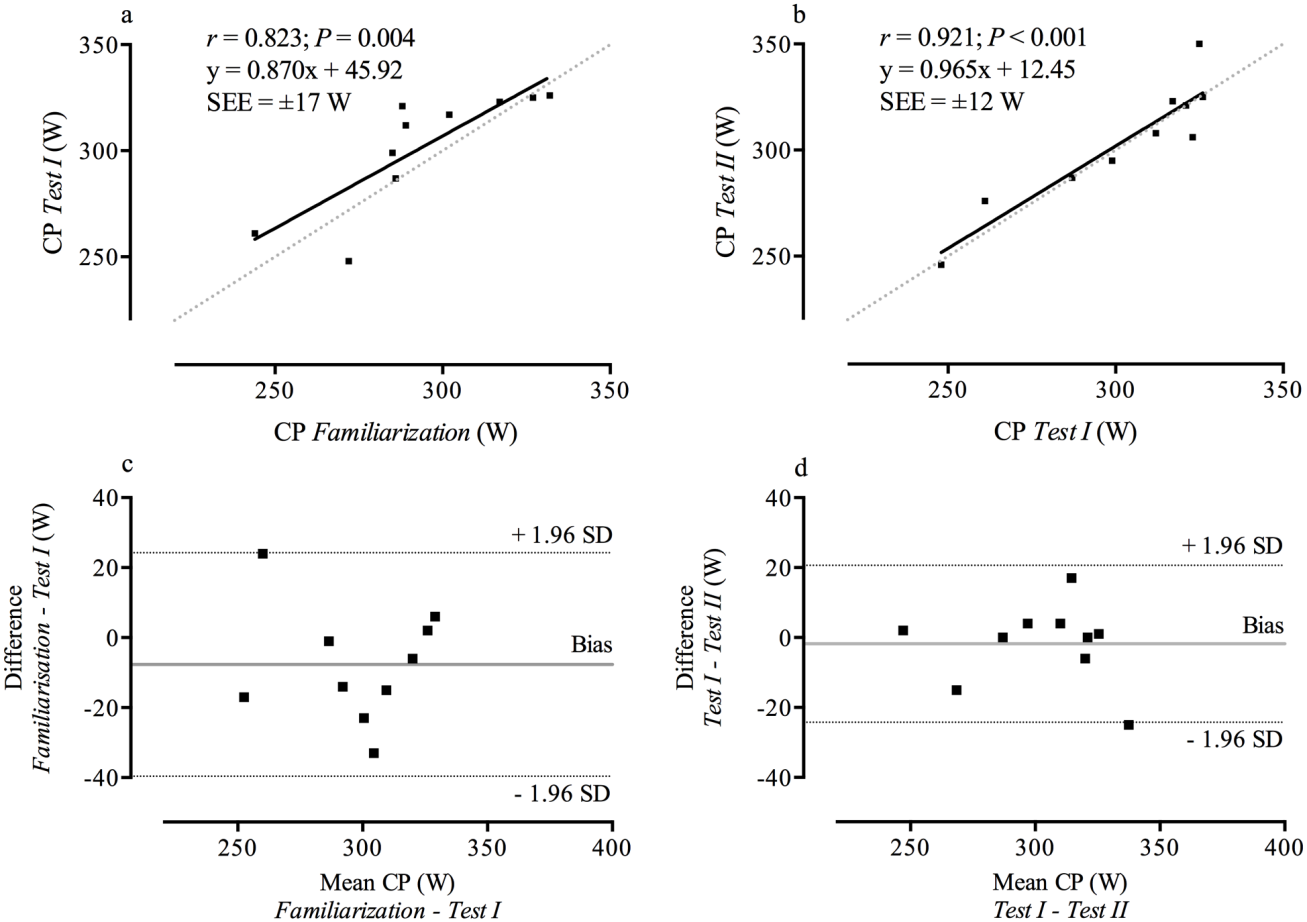
Relationships (panels a and b) and Bland-Altman plots of the differences (panels c and d) between repeated tests of CP. The black solid line represents the linear regression and the grey-dotted line represents the line of identity (panel a and b). The solid grey line represents the mean bias and the dotted black line represent the 95% limits of agreement (panel c and d).

**Fig 2:**
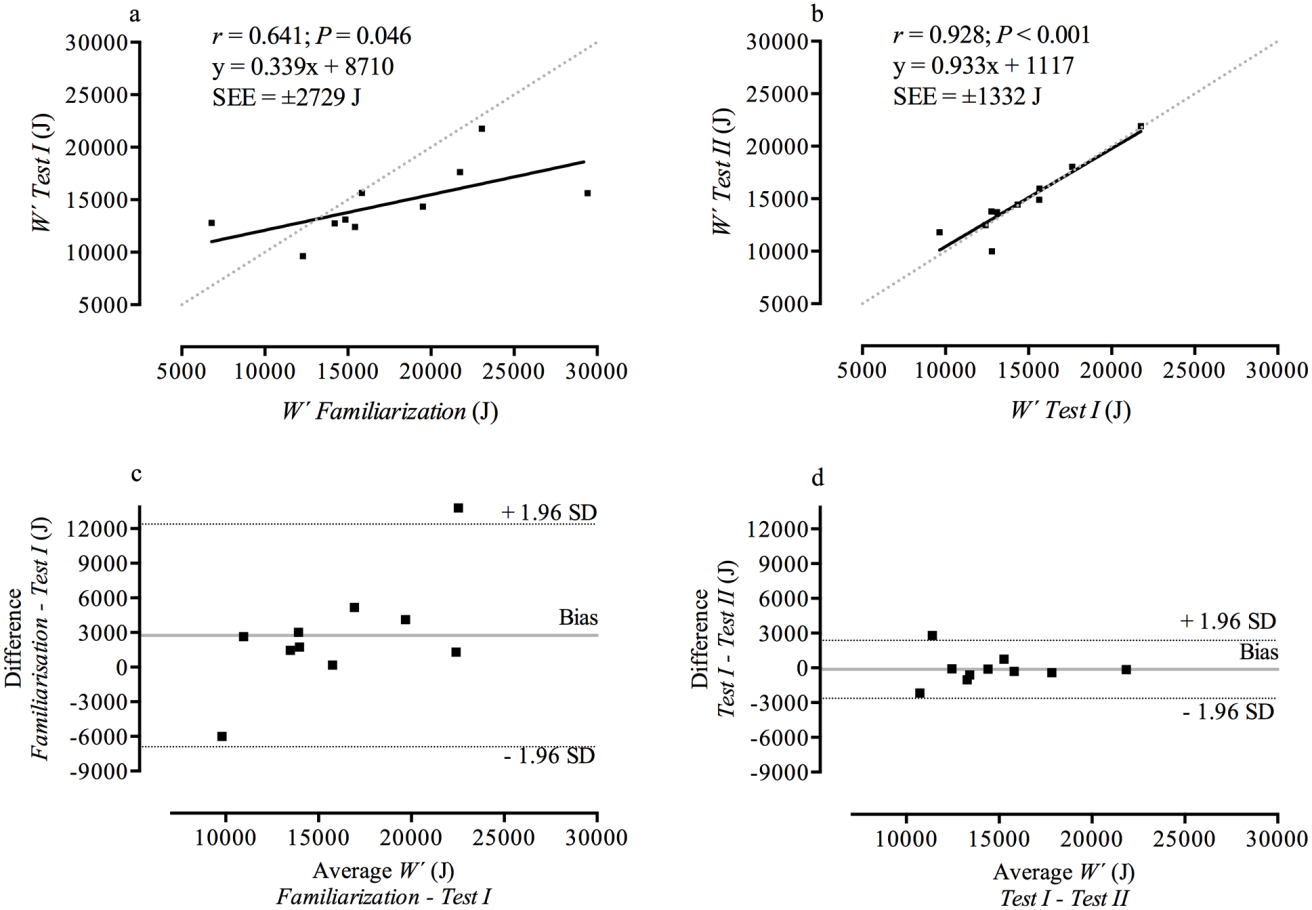
Relationships (panels a and b) and Bland-Altman plots of the differences (panels c and d) between repeated tests of *W´*. The black solid line represents the linear regression and the grey-dotted line represents the line of identity (panel a and b). The solid grey line represents the mean bias and the dotted black line represent the 95% limits of agreement (panel c and d).

Table 1 represents results of *Familiarization*, *Tests I* and *II* (S1 File), Table 2 illustrates data reporting reliability and agreement between repeated tests (Figs 1 and 2), and Table 3 reports the ICC and CoV of individual TT. Figs 1 and 2 illustrate the correlation of CP and *W´* between repeated tests. Between tests non-significant differences were found for CP (*F*_2,18_ = 1.949; *P* = 0.171; $\eta_{p}^{2}=0.178$) and *W´* (*F*_2,18_ = 2.951; *P* = 0.078; $\eta_{p}^{2}=0.247$). Significant differences were found for the absolute SEE for CP (*F*_2,18_ = 10.847; *P* = 0.001; $\eta_{p}^{2}=0.547$) and *W´* (*F*_2,18_ = 10.865; *P* = 0.001; $\eta_{p}^{2}=0.547$) and the relative SEE for CP (*F*_2,18_ = 5.935; *P* = 0.001; $\eta_{p}^{2}=0.549$) and *W´* (*F*_2,18_ = 5.428; *P* = 0.014; $\eta_{p}^{2}=0.376$). Bonferroni post-hoc procedures for the absolute SEE revealed significant differences between *Familiarization* and *Test I* for CP and *W´* (*P* = 0.042 and *d* = 1.20 for both parameters) and between *Familiarization* and *Test II* for CP and *W´* (*P* = 0.008 and *d* = 1.74 for both parameters). No significant differences were found for the absolute SEE for CP (*P* = 0.989 and *d* < 0.01) and the absolute SEE for *W´* (*P* = 0.945 and *d* < 0.01) between *Test I* and *Test II*. Bonferroni post-hoc procedures for the relative SEE revealed significant differences between *Familiarization* and *Test I* and between *Familiarization* and *Test II* for CP only (*P* = 0.043, *d* = 1.04 and *P* = 0.005, *d* = 1.85, respectively), but not for *W´* (*P* = 0.185, *d* = 0.75 and *P* = 0.075, *d* = 0.96, respectively). No significant differences were found for the relative SEE for CP (*P* = 0.850 and *d* = 0.12) and the relative SEE for *W´* (*P* = 0.841 and *d* = 0.12) between *Test I* and *Test II*.

**Table 1:** Results of CP and *W´* and their associated SEE.

	*Familiarization*	*Test I*	*Test II*
CP (W)	294 ± 26	302 ± 28	304 ± 29
*W´* (J)	17316 ± 6340	14972 ± 3052	14710 ± 3368
SEE CP (W)	7.2 ± 3.4	3.8 ± 3.2*	3.1 ± 3.0*
SEE *W´* (J)	2012 ± 963	1060 ± 896*	868 ± 825*
SEE CP (%)	2.4 ± 1.1	1.3 ± 1.1*	1.0 ± 1.0*
SEE *W´* (%)	12.6 ± 7.4´	7.3 ± 6.5	6.0 ± 6.0

CP = Critical Power; *W´* = maximum work above CP; SEE = standard error of the estimate;

*significantly different at *P* < 0.050 from *Familiarization*.

**Table 2:** ICC (95%CL), CoV (95%CL), mean bias and 95% LoA for *W´* and CP.

	*W´* (J)	CP (W)
ICC *Familiarization* vs. *Test I*	0.58 (-0.03 to 0.88)	0.86 (0.53 to 0.96)
ICC *Test I* vs. *Test II*	0.95 (0.80 to 0.99)	0.94 (0.78 to 0.98)
CoV (%) *Familiarization* vs. *Test I*	25.3 (16.8 to 50.9)	4.1 (2.8 to 7.7)
CoV (%) *Test I* vs. *Test II*	8.2 (5.6 to 15.5)	2.6 (1.8 to 4.8)
Bias *Familiarization*–*Test I*	2742	-8
95% LoA	-6899 to 12384	-40 to 24
Bias *Test I*–*Test II*	-135	-2
95% LoA	-2635 to 2366	-24 to 21

ICC = intra-class correlation coefficient; CL = confidence limits; CoV = coefficient of variation; LoA = limits of agreement.

**Table 3:** ICC (95% CL), and CoV (95% CL) for individual TT and test.

	12-min TT	7-min TT	3-min TT
ICC Fam—Test I	0.94 (0.78–0.99)	0.97 (0.88–0.99)	0.95 (0.80–0.99)
CoV Fam—Test I	2.9 (2.0–5.3)	2.0 (1.3–2.6)	3.0 (2.0–5.5)
ICC Test I—Test II	0.95 (0.83–0.99)	0.95 (0.82–0.99)	0.97 (0.87–0.99)
CoV Test I—Test II	2.4 (1.6–4.4)	2.5 (1.7–4.6)	2.5 (1.7–4.6)

Fam = Familiarization

## Discussion

The main novel findings of the present study were that both, CP and *W´* values provide reliable results in a cohort of well-trained athletes after a familiarization trial. Importantly, this is the first study, which demonstrates such a high reliability for the estimates of *W´* (ICC *r* = 0.94). Even though participants were familiar with TT efforts in the field, they produced slightly higher CP estimates (~3.5%) and notably lower *W´* estimates (~13%) after the familiarization trial. Although non-significant differences in the parameter estimates were revealed, the effect size is of a *moderate* order for both parameter estimates, *small* effects were observed between *Familiarization* and *Test I* for CP (*d* = 0.28) and *W´* (*d* = 0.47). The effect sizes for CP and *W´* between *Tests I* and *II* were *trivial* (*d* = -0.04 and *d* = -0.06, respectively). Considering effect sizes seems to be more appropriate when assessing smaller sample sizes and small mean differences [37].

Results demonstrate a notable improvement for ICC and CoV values related to both parameter estimates after familiarization using TT of equal duration (i.e. 12, 7, and 3 min). Recently, it was demonstrated that the high intra-individual variation in parameter estimates can be reduced when using iso-duration TT compared with TTE efforts [12]. The predictive error of *W´* however, remained too high to be used for detecting small training induced changes (i.e. 18.7% [12]). Previous studies suggested that small changes in TTE durations affect *W´* [11, 12] and consequently, using fixed-duration TT can alleviate these negative influences thus increasing reliability of the parameter estimates.

ICC values for CP between *Familiarization* and *Test I* and between *Tests I* and *II* can be interpreted as *moderate* and *highly reliable*, respectively. The CoV for CP notably decreased following the familiarization trial (4.1% vs. 2.6%). But both testing trials were within what is currently acknowledged as an accepted range (i.e. <10% for *W´* [34] and <5% for CP [33]). Our CP results are consistent with studies where reliability of CP was evaluated using TT under laboratory conditions [25] and under field conditions [7]. Karsten et al. [7] found similar ICC values and CoV compared to the present results (ICC *r* = 0.99 and CoV = 2.2%). A recent study by Wright et al. [31] found comparable ICC values (*r* = 0.97–0.99 [31]) and comparable CoV (1.2–1.9% and 8.4% [31] for CP and *W´*, respectively), when using the three minute all-out test (3MT). However, whilst employing TT for the determination of the parameter estimates is a valid method [5], the validity of the 3MT compared to the traditional determination using TTE is poor (i.e. SEE >5% and >26% for CP and *W´*, respectively) [31]. This suggests that the determination of the parameter estimates using multiple TT provides more accurate parameter estimates compared to a single effort, i.e. the 3MT.

While the ICC value for *W´* is interpreted as *poor* between *Familiarization* and *Test I*, it changes to be *highly reliable* between *Tests I* and *II*. Furthermore, the CoV was >10% for *W´* between *Familiarization* and *Test I*, whilst it improved to values that according to Atkinson and Nevill [34] can be interpreted as reliable (i.e. <10%) between *Tests I* and *II*, confirming *W´* to be reliable post familiarization. However, such a high reliability was not present in a field-based study using a similar methodology (ICC *r* = 0.16 and CoV = 46% [7]). Karsten et al. [7] speculated that differences in environmental conditions (e.g. level vs. uphill) might have influenced the results for *W´*. With the exclusion of this factor, our laboratory-based parameter estimates demonstrate a high level of reliability after familiarization (ICC *r* = 0.95). It is therefore suggested that standardized and controlled laboratory conditions alleviate influencing effects on *W´* and consequently result in a higher reliability of the parameter estimate. When using the 3MT, Wright et al. [31] found comparable reliability for *W´* (ICC *r* = 0.94–0.98 and CoV = 5.4–8.4% [31]).

The mean bias of CP and *W´* between *Tests I* and *II* was close to zero after a familiarization session (Figs 1 and 2). Furthermore, the 95% LoA for both parameters showed notably closer LoA after *Familiarization* (Figs 1 and 2) which is consistent with findings using TT in well-trained runners [15]. Galbraith et al. [15] found an improvement of 95% LoA for *W´* from ±80 m to ±45 m (reduction of ~50%) after familiarization, and in the present study a familiarization session resulted in an even greater improvement of the 95% LoA from ± 10,000 J to ± 2,500 J (reduction of ~75%). These results provide evidence of a learning effect even in well-trained cyclists. Similar to the LoA, the SEE became notably smaller for both parameter estimates after a familiarization session (Figs 1 and 2). Our participants were able to provide a more consistent performance thereby reducing SEE by ~30% (CP) and by ~50% (*W´*) after familiarization, also showing the presence of a learning effect. After a familiarization trial, a high agreement of the regression line and the line of identity for both parameter estimates was evident (Figs 1b and 2b). The SEE values between *Tests I* and *II* (±12 W and ±1.3 kJ for CP and *W´*, respectively) are also within day-to-day variations and are lower compared to the recent field-based study by Karsten et al. [7]. The SEE for CP in our study is slightly higher compared to another laboratory-based investigation using TT, however, the SEE for *W´* is similar [25]. It is important to note that Parker-Simpson and Kordi [25] used a different testing methodology by performing the third TT on a different day.

Moreover, Black et al. [24] and Karsten et al. [6] speculated that different pacing patterns (i.e. fast start vs. slow start) between efforts could have affected the determination of CP and *W´*. Galbraith et al. [14] reported a pacing related learning effect in well-trained runners which might be the cause for the low reliability between *Familiarization* and *Test I* in the present study. Contrary to this, Parker-Simpson and Kordi [25] stated the need of two familiarization sessions using TT, but in contrast to the present study, participants were not allowed to change gear ratios during the TT, which lowered ecological validity and likely added to a larger learning effect. Participants in the present study seem to have adapted a reproducible pacing strategy as the mean PO within the first 60 s was not different between respective trials (*P* = 0.561–0.836).

Coakley and Passfield [21] argued that TTE are superior compared to TT as TTE provide a higher mean PO during the longest trial (i.e. ~12 min) compared to TT. However, during the TT in the present study participants were able to select a self-selected pacing strategy with a known end-point and therefore these TT approximated real-world conditions as close as possible. Moreover, the work-rate during the TTE was not constant and participants were able to change PO in a small range [21]. Depending on their research question investigators can take a more informed decision which mode (i.e. TT vs. TTE) to choose. A fast start, as seen during most real-world TT efforts, will stimulate Type III/IV neurons [23], increase the level of pain [22] and thereby the overall exertion, which might result in a reduced PO. However, fluctuations in PO during TT more closely mimic real-world TT and therefore, TT should be preferred to construct the power-duration relationship.

Even though individual TT were highly reliable throughout repeated tests (ICC *r* = 0.94–0.97 and CoV = 2.0–3.0%) (Table 3), lower SEE values of the individual power-duration relationships (i.e. elevated quality of the model) were demonstrated post familiarization (Table 1). Thereafter, SEE values remained low in subsequent tests. The present results support the argument by Karsten et al. [5] who stated that assessing the SEE is an important measure for the quality of the model. The differences in absolute and relative SEE of CP and *W´* between *Familiarization* and *Test I* are of a large effect size, which shows a learning effect and consequently the need for familiarization. Recently, it was suggested that SEE values above recommended limits (i.e. 2% for CP and 10% for *W´* [38, 39]) may affect the parameter estimates [5, 12].

Generally, the reasons for the higher reliability in the current study compared to earlier work could have been threefold: (i) controlled laboratory conditions; (ii) same TT durations across visits; (iii) no differences in pacing strategy after a familiarization session.

A potential limitation of the study was the use of fixed-duration TT. These, whilst arguably carrying a higher ecological validity compared to constant-power TTE, are limited by competitive races commonly using fixed-distances rather than fixed-times. Yet, fixed-duration TT should be preferred to reduce the level of random error and construct the power-duration relationship reproducibly [12]. More research can be suggested to investigate the potential supremacy of fixed-distance TT in the laboratory and the field.

## Conclusion

To reduce the error inherent in testing, present results demonstrate that trained athletes experienced in TT and competition require to be familiarized when determining CP and *W´* using TT in the laboratory. Even though highly reliable results for individual mean TT PO across multiple tests were evident, the quality of the model increased in subsequent testing sessions. Therefore, using TT is valid, reliable, and ecologically valid (i.e. own pacing strategy, change of cadence and gearing). It is consequently suggested that laboratory TT are preferable over TTE efforts and should be considered as a recommended method of best practice when determining CP and *W´*.

## Supporting information

S1 File Individual data for each participant. File contains data for CP, *W´*, and relative and absolute SE for CP and *W´*, respectively.
(XLSX)
